# Association between the C-reactive protein-albumin-lymphocyte index and incident depression in patients with chronic obstructive pulmonary disease: a prospective cohort study from the UK biobank

**DOI:** 10.3389/fmed.2026.1921399

**Published:** 2026-08-28

**Authors:** Yushan Shi, Xianhai Chen, Yan Wang

**Affiliations:** 1 First Clinical Medical College, Shandong University of Traditional Chinese Medicine, Jinan, Shandong, China; 2 Department of Laboratory Medicine, Affiliated Hospital of Shandong University of Traditional Chinese Medicine, Jinan, Shandong, China; 3 Department of Respiratory and Critical Care Medicine, Affiliated Hospital of Shandong University of Traditional Chinese Medicine, Jinan, Shandong, China

**Keywords:** chronic obstructive pulmonary disease, C-reactive protein–albumin–lymphocyte index, depression, non-linear association, prospective cohort study, threshold effect, UK biobank

## Abstract

**Background:**

Depression is a prevalent and underrecognized comorbidity in chronic obstructive pulmonary disease (COPD) that worsens outcomes. The C-reactive protein–albumin–lymphocyte (CALLY) index, a composite immune-nutritional biomarker, has demonstrated prognostic value in various chronic conditions, but its prospective association with incident depression in COPD remains unexplored.

**Methods:**

This prospective cohort study included 40,200 UK Biobank participants with baseline COPD. Cox proportional hazards regression was used to estimate the association between the CALLY index and incident depression, with progressive adjustment across five models. Restricted cubic spline (RCS) curves and two-segment Cox regression was applied to characterize non-linear associations and identify inflection points. Subgroup and sensitivity analyses were conducted to assess robustness.

**Results:**

During a mean follow-up of 13.1 ± 2.8 years, 2,059 incident depression events occurred. In the fully adjusted model, each one-standard deviation (SD) increment in ln-CALLY was associated with a 7% lower risk of depression (HR = 0.93, 95% CI: 0.88–0.97, *P* = 0.002). The highest quartile exhibited an 18% risk reduction vs. the lowest (HR = 0.82, 95% CI: 0.72–0.95, *P* = 0.007). RCS analysis revealed a significant L-shaped relationship (*P* for non-linearity = 0.022). Threshold effect analysis identified an inflection point at CALLY = 17.75; below this threshold, each one-unit increase was associated with a 5% risk reduction (HR = 0.95, 95% CI: 0.90–0.99, *P* = 0.037), whereas above the threshold the association was non-significant. A nominally significant age interaction was observed (*P* = 0.017), though this did not survive Bonferroni correction for multiple testing (adjusted threshold *P* < 0.007). Sensitivity analyses including Fine-Gray competing risk models yielded consistent results.

**Conclusions:**

In COPD patients, the CALLY index exhibits a significant L-shaped association with incident depression risk, characterized by a pronounced protective effect below the identified threshold and a risk plateau above it. These findings suggest that the CALLY index may serve as a clinically accessible tool for depression risk stratification, particularly in younger COPD patients, although the age-specific effect requires confirmation in future studies.

## Introduction

Chronic obstructive pulmonary disease (COPD) is a progressive respiratory condition characterized by persistent airflow limitation and chronic inflammation, representing a major global public health challenge (1–3). COPD affected approximately 392 million people worldwide in 2019, and it has become the third leading cause of death globally, accounting for over 3.2 million deaths annually (4, 5). Beyond its well-established respiratory manifestations, COPD is increasingly recognized as a systemic disease with profound extra-pulmonary consequences. Among these, mental health comorbidities, particularly depression, represent a critical yet often underappreciated dimension of the disease burden (6). Epidemiological studies have consistently demonstrated that the prevalence of depression in COPD patients markedly exceeds that in the general population, with estimates ranging from 20% to 60% depending on the diagnostic criteria and disease severity (7). Depression in COPD not only diminishes quality of life and functional capacity but also exacerbates symptom perception, reduces adherence to pharmacotherapy and pulmonary rehabilitation, and is independently associated with increased hospitalization rates and all-cause mortality (8–10). Despite its clinical significance, depression remains underdiagnosed and undertreated in routine COPD care, partly due to the overlap between somatic symptoms of depression and those of COPD itself, as well as the lack of easily implementable screening tools that integrate biological and clinical risk factors. Therefore, identifying accessible biomarkers that capture the multidimensional pathophysiology underlying COPD-depression comorbidity is of considerable clinical and public health importance.

The C-reactive protein–albumin–lymphocyte (CALLY) index is a novel composite immune nutritional biomarker originally proposed by Iida et al. (11); this index integrates three routinely available laboratory parameters: CRP, reflecting acute-phase systemic inflammation; albumin, serving as a surrogate for nutritional reserve and hepatic synthetic capacity; and lymphocyte count, indicating cellular immune competence (11). Prior investigations have revealed that the CALLY index is a potent indicator in various chronic diseases and malignancies (12–14). Two recent studies using the National Health and Nutrition Examination Survey (NHANES) reported associations between CALLY and depression prevalence in adults with type 2 diabetes mellitus or all-cause mortality among patients with existing depression (15, 16). However, no prior study has examined its predictive value for depression risk specifically in the COPD population.

Therefore, leveraging data from the UK Biobank, this study aimed to investigate the prospective association between the CALLY index and incident depression among individuals with COPD, using a comprehensive analytical framework including Cox proportional hazards models, restricted cubic splines, subgroup analyses, and multiple sensitivity analyses.

## Methods

### Data and study participants

This study utilized data from the UK Biobank, detailed information about this cohort has been reported previously (17). Briefly, between 2006 and 2010, over 500,000 participants aged 40–69 who lived within a roughly 25-mile (40-km) radius of one of 22 assessment centers in Scotland, England and Wales were recruited. At baseline, participants underwent comprehensive assessments to provide a vast amount of information about their health-related factors, and blood, urine, and saliva samples were collected. Health outcomes are systematically tracked through robust linkages to national electronic health records, encompassing mortality data, cancer registry information, hospital admission records, and primary care documentation, which enables the study to capture a wide spectrum of major diseases (18, 19). Participants provided informed consent at baseline assessment, including permission for linkage to national electronic health related datasets. UK Biobank received ethical approval from the North West Multicenter Research Ethics Committee (REC reference: 21/NW/0157). This study was conducted under UK Biobank application number 1263482 (PI: Yushan Shi).

**Figure 1:**
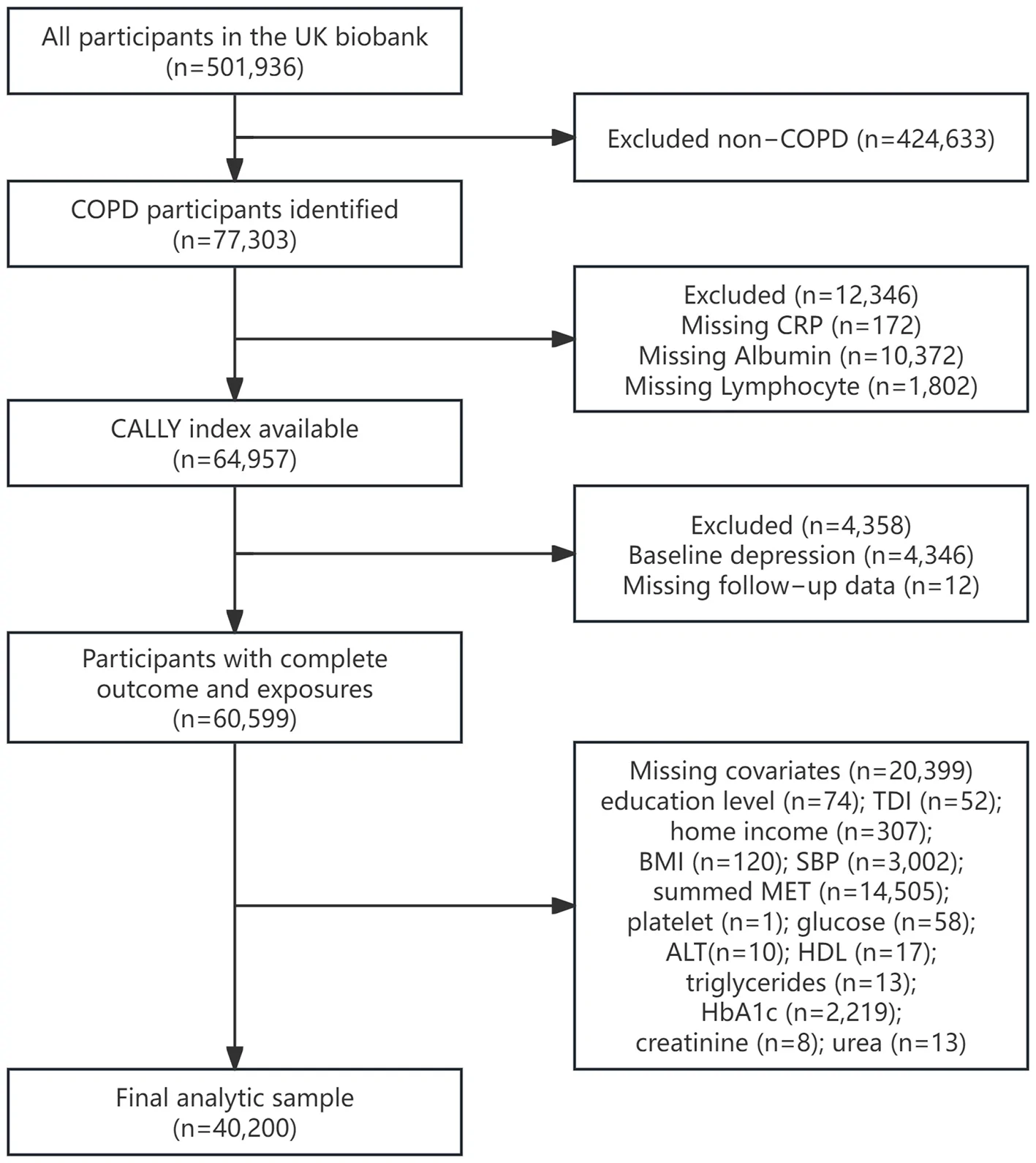
Flowchart of participant selection from the UK Biobank cohort. COPD, chronic obstructive pulmonary disease; CRP, C-reactive protein; TDI, Townsend deprivation index; BMI, body mass index; MET, metabolic equivalent of task; SBP, Systolic blood pressure; ALT, Alanine aminotransferase; HDL, high-density lipoprotein; HbA1c, glycated hemoglobin.

For the present analysis, of the 501,936 participants enrolled in the UK Biobank, we identified 77,303 individuals with COPD at baseline. Participants were excluded if they had missing data for CRP (*n* = 172), albumin (*n* = 10,372), or lymphocyte count (*n* = 1,802), resulting in 64,957 participants with available CALLY index. We further excluded those with baseline depression (n = 4,346) or missing follow-up data (*n* = 12), leaving 60,599 participants with complete outcome and exposure information. After excluding 20,399 participants with missing covariates—including education level (*n* = 74), Townsend Deprivation Index (TDI, *n* = 52), household income (*n* = 307), body mass index (BMI, *n* = 120), systolic blood pressure (*n* = 3,002), summed metabolic equivalent task (MET, *n* = 14,505), platelet count (*n* = 1), glucose (*n* = 58), alanine aminotransferase (ALT, *n* = 10), high-density lipoprotein cholesterol (HDL-c, *n* = 17), triglycerides (*n* = 13), glycated hemoglobin (HbA1c, *n* = 2,219), creatinine (*n* = 8), and urea (*n* = 13)—the final analytic sample comprised 40,200 participants (Figure 1).

### Assessment of CALLY index

The C-reactive protein-albumin-lymphocyte (CALLY) index is a composite immune-nutritional biomarker originally proposed by Iida et al., (11). It integrates three routinely available laboratory parameters: serum C-reactive protein (CRP, mg/dL), albumin (g/dL), and lymphocyte count (/mm^3^). CRP reflects the systemic inflammatory status, albumin serves as a surrogate of nutritional reserve, and lymphocyte count indicates immune competence. Blood tests were conducted utilizing automated hematology analyzers in every survey round, and detailed measurement descriptions are available in the UK Biobank online showcase. In the present study, baseline serum albumin, high-sensitivity CRP, and lymphocyte counts were obtained from the UK Biobank baseline assessment (2006–2010), and the CALLY index was calculated using the original formula proposed by Iida et al. (11): CALLY Index = [Albumin (g/dL) × Lymphocyte count (/mm^3^)] / [CRP (mg/dL) × 104]. Unit conversions were applied as follows: albumin was converted from g/L to g/dL by dividing by 10; CRP was converted from mg/L to mg/dL by dividing by 10; and lymphocyte count was converted from 10^9^/L to /mm^3^ by multiplying by 10^3^.

### COPD participants

Participants with acceptable-quality spirometry data at baseline (Data-Fields 3062 and 3063) were included. FEV1 and FVC were measured using standardized spirometry protocols, and predicted values and z-scores were calculated using the Global Lung Initiative 2012 (GLI-2012) reference equations for the White population (or ethnic-specific equations where appropriate) (20, 21). COPD was defined as post-bronchodilator FEV1/FVC < 0.70 (GOLD criteria). For participants without post-bronchodilator data, pre-bronchodilator measurements were used as a proxy. In addition, participants who reported a doctor's diagnosis of chronic obstructive pulmonary disease, emphysema, or chronic bronchitis (Data-Field 20002: codes 1112, 1113, or 1472) at the baseline assessment were classified as having COPD (22).

### Ascertainment of depression

Incident cases were ascertained using the UK Biobank “First Occurrence” dataset, which records the first documented date of each diagnosis derived from linked hospital inpatient data (Hospital Episode Statistics for England, Scottish Morbidity Records, and Patient Episode Database for Wales), primary care records, and death registries. Cases were identified using International Classification of Diseases, 10th Revision (ICD-10) codes F32 (depressive episode) and F33 (recurrent depressive disorder) (23). This approach captures both first-episode and recurrent depression diagnosed in secondary care and coded primary care settings. Participants who reported a physician diagnosis of depression at the baseline assessment (Data-Field 20127) were excluded to ensure that only incident (new-onset) cases were captured. Follow-up time was calculated from the date of baseline assessment to the date of first depression diagnosis, death, loss to follow-up, or the censoring date of the hospital records (whichever occurred first).

### Covariates

Covariates were selected a priori based on known or suspected associations with CALLY and COPD, as informed by prior literature and previous UK Biobank analyses (16, 24–26). Demographic variables included age, sex, self-reported ethnic background (White, and Not-White), assessment centers (England, Scotland, and Wales), household income and education level (college or university degree, any school degree, vocational qualifications, and others). Socioeconomic status was assessed using the Townsend deprivation index (an area-based measure of socioeconomic deprivation (27). Lifestyle behaviors comprised smoking status (never, former, and current), alcohol consumption status (never, former, and current), sleep duration, summed metabolic equivalent task (MET) minutes per week for all activity. Physical activity was quantified as total metabolic equivalent task (MET) minutes per week for all activities, including walking, moderate, and vigorous activity (28).

Anthropometric and vital sign measurements included body mass index (BMI), it was categorized as underweight (<18.5 kg/m^2^), normal weight (18.5–24.9 kg/m^2^), overweight (25.0–29.9 kg/m^2^), and obese (≥30 kg/m^2^) (29), systolic blood pressure (SBP). Pulmonary function was assessed using forced vital capacity (FVC), forced expiratory volume in 1 second (FEV1), and the FEV1/FVC ratio (27). Blood biochemical parameters included white blood cell count (10^9^/L), platelet count (10^9^/L), hemoglobin concentration (g/dL), glucose (mmol/L), alanine aminotransferase (ALT, U/L), creatinine (umol/L), glycated hemoglobin (HbA1c, mmol/L), high-density lipoprotein cholesterol (HDL-C, mmol/L), triglycerides (mmol/L), and urea (mmol/L). Comorbidities were defined as self-reported or physician-diagnosed conditions and included asthma, diabetes, and hypertension (all coded as yes/no).

### Statistical analyses

Owing to the skewed distribution of the raw CALLY index, values were natural-log-transformed (ln-CALLY) and analyzed both as a continuous variable [per one-unit increment and per standard deviation (SD) increment] and as a categorical variable divided into quartiles (Q1–Q4, with Q1 as the reference). Normally distributed continuous variables were expressed presented as mean± SD, and skewed continuous variables were described as median [inter quartile range (IQR)]. Categorical variables were presented as frequencies (%). The Chi-square (categorical variables), One-Way ANOVA test (normal distribution), or Kruskal-Wallis H test (skewed distribution) was used to test for differences among different ln-CALLY groups.

Multivariable Cox proportional hazards regression was used to estimate hazard ratios (HRs) and 95% confidence intervals (CIs) for the association between CALLY and incident depression. The proportional hazards assumption was tested using Schoenfeld residuals and was satisfied for all final models. Variance inflation factors (VIF) were examined to assess multicollinearity. VIF values (>5) indicating moderate-to-strong collinearity, all remaining covariates yielded VIF values below 5, indicating no problematic multicollinearity. Model 1: unadjusted. Model 2: adjusted for age, sex, ethnic background, education level, household income, TDI, and assessment. Model 3: additionally adjusted for BMI, smoking status, alcohol drinker status, summed MET, Sleep duration, SBP, FVC, and FEV1. Model 4: additionally adjusted for WBC, platelet, hemoglobin, glucose, ALT, creatinine, HbA1c, HDL-C, triglycerides, and Urea. Model 5: additionally adjusted for history of asthma, diabetes, hypertension.

Kaplan-Meier survival curves were generated to visualize the cumulative incidence of depression across CALLY quartiles, and between-group differences were compared using the log-rank test.

Restricted cubic spline (RCS) curves with four knots (placed at the 5th, 35th, 65th, and 95th percentiles) were fitted within the fully adjusted Cox model to examine potential non-linear dose-response relationships between CALLY and depression risk. In order to minimize the impact of outliers, only 99.9% of the data is shown. A threshold effect analysis was additionally performed using a two-segment Cox regression model on the ln-CALLY scale; the inflection point was identified by a recursive algorithm, and HRs were estimated separately below and above this cut-off. A log-likelihood ratio test was used to compare the piecewise model with the single linear model. It should be noted that the identified breakpoint is a data-driven statistical estimate specific to the present cohort and should not be interpreted as a clinically validated cut-off value.

Subgroup analyses were conducted by age (<65 vs. ≥65 years), sex (female vs. male), ethnic background (White vs. non-White), smoking status (never, former, current), BMI category (<25, 25.0–29.9, ≥30 kg/m^2^), diabetes (yes vs. no) and hypertension (yes vs. no). Interaction terms between ln-CALLY and each stratification variable were incorporated into the regression models, and the *P* values for interaction were used to evaluate effect modification. Given that seven interaction tests were performed, a Bonferroni-corrected significance threshold of *P* < 0.05/7 ≈ 0.007 was applied. All subgroup analyses should be interpreted as exploratory.

Sensitivity analyses were conducted to evaluate the robustness of the primary findings. First, to minimize potential reverse causation, we excluded participants with less than 2 years of follow-up (*n* = 468) and repeated the Cox regression analyses in the remaining cohort (*n* = 39,732). Second, to address potential bias from missing covariate data, we performed multiple imputation by chained equations (MICE) with 5 imputed datasets for the 60,599 participants with complete exposure and outcome information. Imputation models included all variables in the fully adjusted model as predictors, and results were pooled using Rubin's rules (30). Third, to account for the competing risk of death, we performed a Fine-Gray subdistribution hazard model treating all-cause mortality as the competing event for incident depression. Fourth, to assess potential selection bias from co-existing respiratory conditions, we conducted a sensitivity analysis excluding participants with concomitant bronchiectasis (Data-Field 20002: code 1114), asthma (code 1111), or bronchitis (code 1412). Fifth, to evaluate the added value of the composite CALLY index over its individual components, we compared model discrimination using the area under the receiver operating characteristic curve (AUC). Continuous net reclassification improvement (NRI) and integrated discrimination improvement (IDI) were calculated to assess the incremental predictive value of CALLY vs. CRP alone.

The consistency of effect estimates across these sensitivity analyses was assessed relative to the primary analytic sample.

All statistical analyses were performed using R software (version 4.2.2; R Foundation for Statistical Computing, Vienna, Austria) and the Free Statistics analysis platform (version 2.3.1, Beijing, China). A two-sided *P* value < 0.05 was considered statistically significant.

## Results

### Characteristics of the participants

Table 1 summarizes the baseline characteristics of 40,200 COPD participants according to CALLY quartiles. The cohort had a mean age of 59.0 years and 57.7% were male. Participants with lower CALLY index (Q1) were older, more likely to be male, and exhibited lower socioeconomic status, characterized by higher Townsend Deprivation Index, lower household income, and fewer college degrees compared with those in Q4 (all *P* < 0.001). Lifestyle profiles also differed markedly: Q1 had the highest prevalence of obesity (33.2% vs. 7.0% in Q4), current smoking (19.9% vs. 14.0%), and physical inactivity (19.9% vs. 11.1%), alongside higher systolic blood pressure. Reflecting the composite nature of CALLY, CRP levels decreased substantially from Q1 to Q4 (7.4 to 0.5 mg/L), whereas albumin and lymphocyte count increased. Comorbidity burden was consistently higher in lower quartiles, including asthma, diabetes, and hypertension (all *P* < 0.001). Notably, the incidence of depression during follow-up decreased progressively across CALLY quartiles, from 6.4% in Q1 to 4.0% in Q4 (*P* < 0.001).

**Table 1:** Baseline characteristics of COPD individuals according to CALLY index.

Variables	Total (*n* = 40,200)	Q1 (<2.91) (*n* = 10,050)	Q2 (2.91–6.10) (*n* = 10,050)	Q3 (6.10–12.52) (*n* = 10,050)	Q4 (≥12.52) (*n* = 10,050)	*p*
Age, years	59.0 ± 7.6	60.3 ± 7.1	59.6 ± 7.4	58.8 ± 7.6	57.2 ± 8.0	<0.001
Sex, *n* (%)						<0.001
Female	16,999 (42.3)	4,213 (41.9)	4,123 (41)	4,117 (41)	4,546 (45.2)	
Male	23,201 (57.7)	5,837 (58.1)	5,927 (59)	5,933 (59)	5,504 (54.8)	
Ethnic background, *n* (%)						<0.001
Non-White	3,514 (8.7)	790 (7.9)	862 (8.6)	898 (8.9)	964 (9.6)	
White	36,686 (91.3)	9,260 (92.1)	9,188 (91.4)	9,152 (91.1)	9,086 (90.4)	
Assessment centers, *n* (%)						<0.001
England	36,864 (91.7)	9,097 (90.5)	9,228 (91.8)	9,263 (92.2)	9,276 (92.3)	
Scotland	1,495 (3.7)	437 (4.3)	345 (3.4)	360 (3.6)	353 (3.5)	
Wales	1,841 (4.6)	516 (5.1)	477 (4.7)	427 (4.2)	421 (4.2)	
Townsend Deprivation Index	−1.2 ± 3.1	−0.8 ± 3.3	−1.2 ± 3.1	−1.4 ± 3.0	−1.4 ± 3.0	<0.001
Average total household income, *n* (%)						<0.001
Less than 18,000	14,261 (35.5)	4,472 (44.5)	3,665 (36.5)	3,252 (32.4)	2,872 (28.6)	
18,000–31,000	9,652 (24.0)	2,424 (24.1)	2,509 (25)	2,440 (24.3)	2,279 (22.7)	
31,000–52,000	8,447 (21.0)	1,753 (17.4)	2,109 (21)	2,230 (22.2)	2,355 (23.4)	
52,000–100,000	6,175 (15.4)	1,134 (11.3)	1,408 (14)	1,659 (16.5)	1,974 (19.6)	
Greater than 100,0001	1,665 (4.1)	267 (2.7)	359 (3.6)	469 (4.7)	570 (5.7)	
Education level, *n* (%)						<0.001
College or university degree	12,627 (31.4)	2,397 (23.9)	2,918 (29)	3,279 (32.6)	4,033 (40.1)	
Any school degree	14,158 (35.2)	3,394 (33.8)	3,560 (35.4)	3,672 (36.5)	3,532 (35.1)	
Vocational qualifications	5,155 (12.8)	1,440 (14.3)	1,323 (13.2)	1,283 (12.8)	1,109 (11)	
Others	8,260 (20.5)	2,819 (28)	2,249 (22.4)	1,816 (18.1)	1,376 (13.7)	
BMI, *n* (%)						<0.001
Underweight (<18.5 kg/m^2^)	322 (0.8)	36 (0.4)	41 (0.4)	70 (0.7)	175 (1.7)	
Normal weight (18.5–24.9 kg/m^2^)	15,172 (37.7)	2,515 (25)	2,968 (29.5)	3,945 (39.3)	5,744 (57.2)	
Overweight (25.0–29.9 kg/m^2^)	16,843 (41.9)	4,165 (41.4)	4,705 (46.8)	4,542 (45.2)	3,431 (34.1)	
Obese (≥30 kg/m^2^)	7,863 (19.6)	3,334 (33.2)	2,336 (23.2)	1,493 (14.9)	700 (7)	
Sleep duration, hour/day	7.2 ± 1.2	7.2 ± 1.3	7.2 ± 1.2	7.1 ± 1.1	7.1 ± 1.1	0.003
Smoking status, ***n*** (%)						<0.001
Never	17,617 (43.8)	3,588 (35.7)	4,211 (41.9)	4,681 (46.6)	5,137 (51.1)	
Former	15,987 (39.8)	4,458 (44.4)	4,160 (41.4)	3,859 (38.4)	3,510 (34.9)	
Current	6,596 (16.4)	2,004 (19.9)	1,679 (16.7)	1,510 (15)	1,403 (14)	
Alcohol drinker status, *n* (%)						<0.001
Never	1,471 (3.7)	408 (4.1)	399 (4)	338 (3.4)	326 (3.2)	
Former	1,556 (3.9)	464 (4.6)	376 (3.7)	341 (3.4)	375 (3.7)	
Current	37,173 (92.5)	9,178 (91.3)	9,275 (92.3)	9,371 (93.2)	9,349 (93)	
Summed MET, minutes/week, *n* (%)						<0.001
< 500	6,052 (15.1)	1,998 (19.9)	1,588 (15.8)	1,346 (13.4)	1,120 (11.1)	
≥500	34,148 (84.9)	8,052 (80.1)	8,462 (84.2)	8,704 (86.6)	8,930 (88.9)	
SBP, mmHg	141.3 ± 19.7	144.2 ± 20.1	143.0 ± 19.7	141.1 ± 19.4	137.2 ± 19.1	<0.001
Lymphocyte count, 10^9^/L	2.0 ± 1.3	1.8 ± 0.6	1.9 ± 0.6	2.0 ± 0.7	2.2 ± 2.4	<0.001
WBC, 10^9^/L	7.1 ± 2.2	7.5 ± 2.1	7.1 ± 1.8	6.9 ± 1.8	6.8 ± 3.0	<0.001
Platelet, 10^9^/L	250.1 ± 60.2	258.2 ± 67.1	251.1 ± 58.6	246.9 ± 57.1	244.0 ± 56.5	<0.001
Albumin, g/L	45.0 ± 2.6	44.0 ± 2.7	44.9 ± 2.5	45.3 ± 2.5	45.8 ± 2.5	<0.001
CRP, mg/L	2.7 ± 4.7	7.4 ± 7.5	2.1 ± 0.8	1.1 ± 0.4	0.5 ± 0.2	<0.001
Glucose, mmol/L	5.1 ± 1.2	5.3 ± 1.5	5.1 ± 1.2	5.1 ± 1.1	5.0 ± 1.0	<0.001
ALT, U/L	19.8 (15.4, 26.4)	20.4 (15.6, 27.7)	20.7 (16.0, 27.6)	19.9 (15.4, 26.4)	18.4 (14.6, 24.0)	<0.001
Creatinine, umol/L	74.5 ± 20.0	76.2 ± 28.3	74.8 ± 17.8	74.3 ± 16.5	72.8 ± 14.5	<0.001
HbA1c, mmol/L	36.4 ± 7.0	37.7 ± 9.3	36.6 ± 6.7	36.0 ± 5.9	35.4 ± 5.4	<0.001
HDL cholesterol, mmol/L	1.4 ± 0.4	1.4 ± 0.4	1.4 ± 0.4	1.5 ± 0.4	1.5 ± 0.4	<0.001
Triglycerides, mmol/L	1.5 (1.0, 2.1)	1.6 (1.2, 2.3)	1.6 (1.1, 2.3)	1.5 (1.0, 2.1)	1.2 (0.9, 1.8)	<0.001
Urea, mmol/L	5.5 ± 1.5	5.6 ± 1.7	5.6 ± 1.4	5.5 ± 1.3	5.4 ± 1.3	<0.001
Hemoglobin, g/dL	14.4 ± 1.2	14.3 ± 1.3	14.5 ± 1.2	14.4 ± 1.2	14.3 ± 1.2	<0.001
FVC, L	3.9 ± 1.4	3.6 ± 1.3	3.8 ± 1.4	3.9 ± 1.5	4.1 ± 1.5	<0.001
FEV1, L	2.5 ± 0.8	2.3 ± 0.8	2.4 ± 0.8	2.5 ± 0.8	2.6 ± 0.9	<0.001
Asthma, *n* (%)						<0.001
No	30,617 (76.2)	7,283 (72.5)	7,590 (75.5)	7,764 (77.3)	7,980 (79.4)	
Yes	9,583 (23.8)	2,767 (27.5)	2,460 (24.5)	2,286 (22.7)	2,070 (20.6)	
Diabetes, *n* (%)						<0.001
No	38,036 (94.6)	9,331 (92.8)	9,527 (94.8)	9,530 (94.8)	9,648 (96)	
Yes	2,164 (5.4)	719 (7.2)	523 (5.2)	520 (5.2)	402 (4)	
Hypertension, *n* (%)						<0.001
No	28,277 (70.3)	6,219 (61.9)	6,899 (68.6)	7,255 (72.2)	7,904 (78.6)	
Yes	11,923 (29.7)	3,831 (38.1)	3,151 (31.4)	2,795 (27.8)	2,146 (21.4)	
Depression event, No. (%)						<0.001
No	38,141 (94.9)	9,408 (93.6)	9,488 (94.4)	9,601 (95.5)	9,644 (96)	
Yes	2,059 (5.1)	642 (6.4)	562 (5.6)	449 (4.5)	406 (4)	
Follow up time, years	13.1 ± 2.8	12.5 ± 3.4	13.1 ± 2.8	13.3 ± 2.5	13.4 ± 2.3	<0.001

Data are presented as mean (SD), median (IQR), or No. (%). Quartile groups (Q1-Q4) are based on CALLY values, with Q1 representing the lowest and Q4 the highest. P values were derived from one-way ANOVA for continuous variables, Kruskal-Wallis test for skewed variables, and chi-square test for categorical variables. COPD, chronic obstructive pulmonary disease; CALLY, C-reactive protein-albumin-lymphocyte index; TDI, Townsend deprivation index; BMI, body mass index; MET, metabolic equivalent of task; SBP, Systolic blood pressure; WBC, White blood cell; CRP, C-reactive protein; ALT, Alanine aminotransferase; HbA1c, glycated hemoglobin; HDL, high-density lipoprotein; FVC, forced vital capacity; FEV1, forced expiratory volume in 1 second; IQR, interquartile range; SD, standard deviation.

### Association between the CALLY index and incident depression

**Figure 2:**
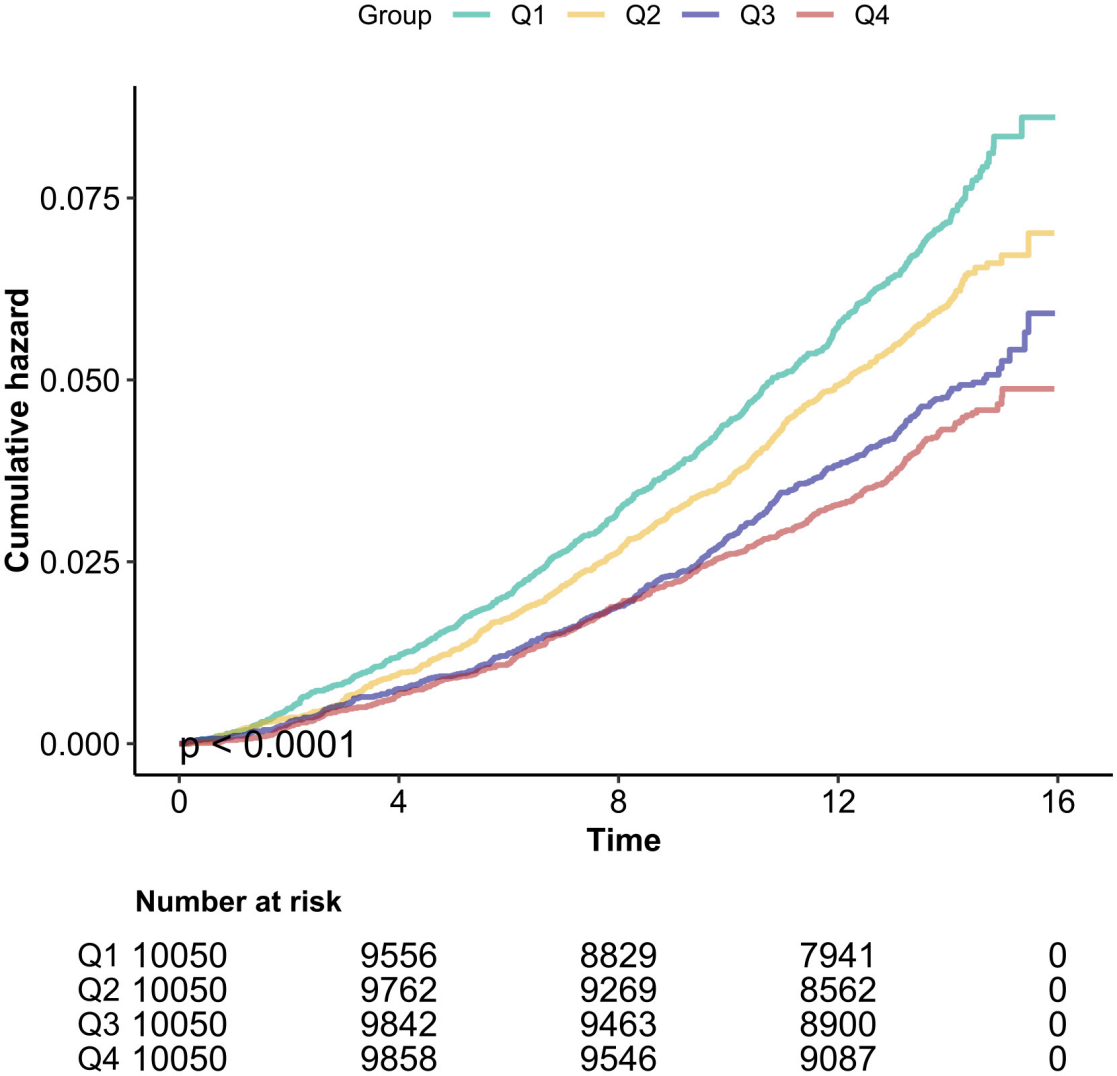
Cumulative incidence of depression by quartiles of CALLY index. COPD, chronic obstructive pulmonary disease; CALLY, C-reactive protein–albumin–lymphocyte index; Q1, first quartile; Q2, second quartile; Q3, third quartile; Q4, fourth quartile.

The cumulative incidence of depression across CALLY quartiles was shown in Figure 2. Throughout the follow-up period, participants in Q4 consistently exhibited the lowest cumulative event rates, whereas those in Q1 had the highest (log-rank *P* < 0.0001).

Table 2 shows the association between CALLY index and incident depression in COPD participants. Participants were categorized into CALLY quartiles: Q1(<2.91), Q2 (2.91–6.10), Q3 (6.10–12.52), and Q4 (≥12.52), based on the recalculated Iida-equivalent CALLY values. In the unadjusted model, each 1-SD increase in ln-CALLY was associated with a 18% lower risk of depression (HR 0.82, 95% CI 0.78–0.85). After progressive multivariable adjustment, this association was attenuated but remained statistically significant in the fully adjusted model (Model 5: HR 0.93, 95% CI 0.88–0.97, *P* = 0.002). When analyzed by quartiles, a graded inverse association was observed. After full adjustment for sociodemographic, lifestyle, clinical, and comorbidity variables, the association for Q2 became non-significant (HR 0.98, 95% CI 0.87–1.10, *P* = 0.73), whereas Q3 (HR 0.83, 95% CI 0.73–0.95, *P* = 0.006) and Q4 (HR 0.82, 95% CI 0.72–0.95, *P* = 0.007) retained significant protective effects. The linear trend across quartiles remained significant in all models (*P* for trend = 0.001 in Model 5), indicating a robust inverse dose–response relationship between higher CALLY index and lower depression incidence in COPD patients.

**Table 2:** Association between CALLY index and depression incidence in COPD participants.

Variable	Model 1		Model 2		Model 3		Model 4		Model 5	
	HR (95%CI)	*p*	HR (95%CI)	*p*	HR (95%CI)	*p*	HR (95%CI)	*p*	HR (95%CI)	*p*
ln-CALLY	0.83 (0.80~0.87)	<0.001	0.88 (0.84~0.91)	<0.001	0.93 (0.89~0.97)	0.001	0.93 (0.89~0.97)	0.001	0.93 (0.89~0.98)	0.002
Per SD in ln-CALLY	0.82 (0.78~0.85)	<0.001	0.86 (0.83~0.90)	<0.001	0.92 (0.87~0.96)	0.001	0.92 (0.88~0.97)	0.001	0.93 (0.88~0.97)	0.002
CALLY quartiles										
Q1 (<2.91)	1(Ref)		1(Ref)		1(Ref)		1(Ref)		1(Ref)	
Q2 (2.91–6.10)	0.83 (0.75~0.93)	0.002	0.90 (0.80~1.00)	0.056	0.95 (0.85~1.07)	0.433	0.97 (0.86~1.09)	0.633	0.98 (0.87~1.10)	0.73
Q3 (6.10–12.52)	0.65 (0.58~0.74)	<0.001	0.74 (0.65~0.83)	<0.001	0.81 (0.71~0.92)	0.001	0.83 (0.73~0.94)	0.005	0.83 (0.73~0.95)	0.006
Q4 (≥12.52)	0.58 (0.52~0.66)	<0.001	0.69 (0.60~0.78)	<0.001	0.81 (0.70~0.93)	0.003	0.82 (0.71~0.94)	0.006	0.82 (0.72~0.95)	0.007
*P* for trend		<0.001		<0.001		<0.001		0.001		0.001

Model 1: unadjusted. Model 2: adjusted for age, sex, ethnic background, education level, household income, TDI, and assessment. Model 3: additionally adjusted for BMI, smoking status, alcohol drinker status, summed MET, Sleep duration, SBP, FVC, and FEV1. Model 4: additionally adjusted for WBC, platelet, hemoglobin, glucose, ALT, creatinine, HbA1c, HDL-C, triglycerides, and Urea. Model 5: additionally adjusted for history of asthma, diabetes, hypertension. COPD, chronic obstructive pulmonary disease; CALLY, C-reactive protein-albumin-lymphocyte index; TDI, Townsend deprivation index; BMI, body mass index; MET, metabolic equivalent of task; SBP, Systolic blood pressure; WBC, White blood cell; CRP, C-reactive protein; ALT, Alanine aminotransferase; HbA1c, glycated hemoglobin; HDL, high-density lipoprotein; FVC, forced vital capacity; FEV1, forced expiratory volume in 1 second.

### Non-linear association and threshold effect analysis

**Figure 3:**
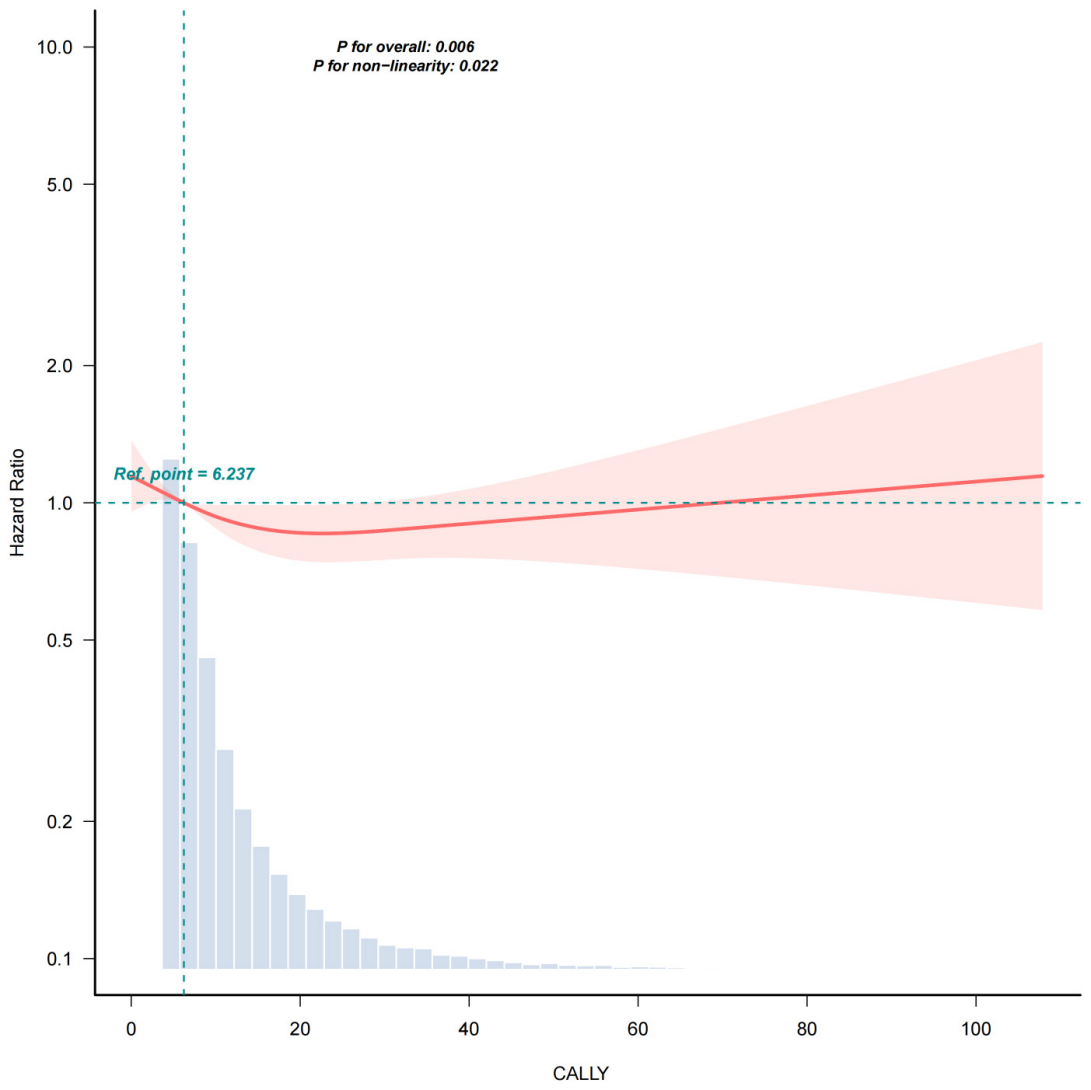
Non-linear dose–response relationship between CALLY and incident depression. The solid red line represents the estimated hazard ratio, and the shaded area indicates the 95% confidence interval. The histogram shows the distribution of CALLY values in the study population. The vertical dashed line marks the reference point (median CALLY = 6.237, HR = 1.0). adjusted for age, sex, ethnic background, education level, household income, TDI, assessment, BMI, smoking status, alcohol drinker status, summed MET, Sleep duration, SBP, FVC, FEV1, WBC, platelet, hemoglobin, glucose, ALT, creatinine, HbA1c, HDL-C, triglycerides, Urea, history of asthma, diabetes, hypertension. Only 99.9% of the data is displayed. COPD, chronic obstructive pulmonary disease; CALLY, C-reactive protein-albumin-lymphocyte index; TDI, Townsend deprivation index; BMI, body mass index; MET, metabolic equivalent of task; SBP, Systolic blood pressure; FVC, forced vital capacity; FEV1, forced expiratory volume in 1 second; WBC, White blood cell; CRP, C-reactive protein; ALT, alanine aminotransferase; HbA1c, glycated hemoglobin; HDL, high-density lipoprotein.

As shown in Figure 3, the restricted cubic spline curve exhibited a L-shaped pattern between CALLY index and incident depression (*P* for non-linearity = 0.022). This non-linear pattern was further corroborated by threshold effect analysis (Table 3). Using a two-piecewise linear regression model, the estimated breakpoint was 17.75. Below this threshold, each unit increase in ln-CALLY was associated with a 5% reduction in depression risk (HR 0.95, 95% CI 0.90–0.99, *P* = 0.037). Above the breakpoint, the association was no longer significant (HR 1.14, 95% CI 0.81–1.60, *P* = 0.449), and the likelihood ratio test comparing the two-segment model with the linear model was significant (*P* = 0.013).

**Table 3:** Threshold effect analysis of the association between CALLY and depression incidence in COPD participants.

Item	*n*	HR (95%CI)	*P* value
CALLY < 17.75 (ln-CALLY = 2.87)	33,975	0.95 (0.90~0.99)	0.037
CALLY≥17.75	6,184	1.14 (0.81~1.60)	0.449
Likelihood Ratio test			0.013

Adjusted for age, sex, ethnic background, education level, household income, TDI, assessment, BMI, smoking status, alcohol drinker status, summed MET, Sleep duration, SBP, FVC, FEV1, WBC, platelet, hemoglobin, glucose, ALT, creatinine, HbA1c, HDL-C, triglycerides, Urea, history of asthma, diabetes, hypertension. COPD, chronic obstructive pulmonary disease; CALLY, C-reactive protein-albumin-lymphocyte index; TDI, Townsend deprivation index; BMI, body mass index; MET, metabolic equivalent of task; SBP, Systolic blood pressure; FVC, forced vital capacity; FEV1, forced expiratory volume in 1 second; WBC, White blood cell; CRP, C-reactive protein; ALT, Alanine aminotransferase; HbA1c, glycated hemoglobin; HDL, high-density lipoprotein. Only 99.9% of the data is displayed.

### Subgroup analyses

**Figure 4:**
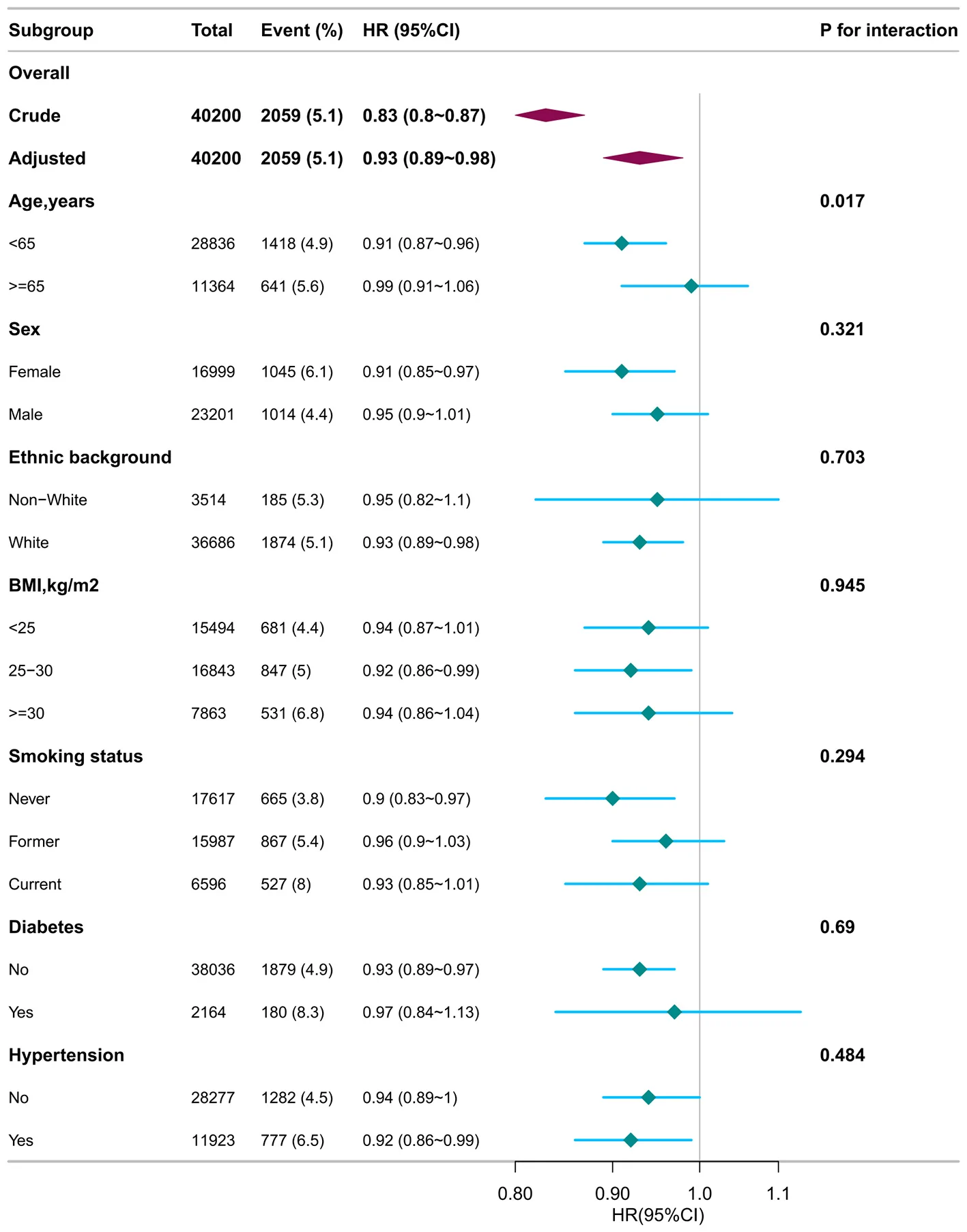
Subgroup analyses of the association between CALLY and incident depression. Except for subgroup itself, model was adjusted for age, sex, ethnic background, education level, household income, TDI, assessment, BMI, smoking status, alcohol drinker status, summed MET, Sleep duration, SBP, FVC, FEV1, WBC, platelet, hemoglobin, glucose, ALT, creatinine, HbA1c, HDL-C, triglycerides, Urea, history of asthma, diabetes, hypertension. COPD, chronic obstructive pulmonary disease; CALLY, C-reactive protein-albumin-lymphocyte index; TDI, Townsend deprivation index; BMI, body mass index; MET, metabolic equivalent of task; SBP, Systolic blood pressure; FVC, forced vital capacity; FEV1, forced expiratory volume in 1 second; WBC, White blood cell; CRP, C-reactive protein; ALT, Alanine aminotransferase; HbA1c, glycated hemoglobin; HDL, high-density lipoprotein.

Stratified analyses by age, sex, ethnic background, smoking status, BMI category, diabetes and hypertension were presented in Figure 4. Across all subgroups, higher ln-CALLY remained significantly associated with a lower risk of incident depression in the fully adjusted model, and the direction of association was consistent. A significant interaction was observed only for age (*p* for interaction = 0.017), whereby the protective effect of CALLY was evident in participants younger than 65 years (HR 0.91, 95% CI 0.87–0.96) but not in those aged 65 years or older (HR 0.99, 95% CI 0.91–1.06).

### Sensitivity analyses

After excluding participants with less than 2 years of follow-up (*n* = 39,732), the association between ln-CALLY and incident depression remained significant in the fully adjusted model (HR 0.94, 95% CI 0.90–0.98, *P* = 0.005), with Q3 and Q4 showing consistent protective effects (Supplementary Table S1). Multiple imputation for missing covariates in the complete exposure-outcome cohort (*n* = 60,599) yielded similar results, with ln-CALLY maintaining a significant inverse association (Model 5: HR 0.94, 95% CI 0.90–0.98, *P* = 0.003) and Q3 and Q4 remaining significant across all adjusted models (Supplementary Table S2). In the Fine-Gray competing risk model treating all-cause mortality as the competing event, each one unit increase in ln-CALLY was associated with a subdistribution HR of 0.96 (95% CI: 0.92–0.99, *P* = 0.035) for incident depression (Supplementary Table S3). The Fine-Gray results were consistent with the primary Cox regression findings. After excluding participants with concomitant bronchiectasis, asthma, or bronchitis (Supplementary Table S4, *n* = 30,526), the association between ln-CALLY and incident depression remained consistent with the primary analysis (Model 5: HR = 0.94, 95% CI: 0.89–0.99, *P* = 0.015), with significant protective effects persisting in the highest quartiles (Q3: HR = 0.80, *P* = 0.005; Q4: HR = 0.81, *P* = 0.01).

In the comparison of CALLY vs. individual components, the baseline AUC for CALLY was 56.77% (95% CI: 55.51–58.04%), which was numerically higher than CRP (55.72%), albumin (53.55%), and lymphocyte count (54.06%) (Supplementary Figure S1). The continuous net reclassification improvement (NRI) indicated that CALLY significantly improved risk reclassification compared with CRP alone (Supplementary Table S5, NRI = 0.0844, 95% CI: 0.0415–0.1272, *P* = 0.00011), whereas the integrated discrimination improvement (IDI) was directionally positive but not statistically significant (IDI = 0.0001, 95% CI: 0–0.0002, *P* = 0.077).

The quartile-specific effect estimates and trend tests were highly consistent with the primary analysis, indicating that the observed association was not substantially influenced by reverse causation or missing data patterns.

## Discussion

This large prospective cohort study of 40,200 COPD patients from the UK Biobank demonstrated that a higher CALLY index was independently associated with a lower risk of incident depression over a mean follow-up of 13.1 years. The association exhibited a significant non-linear pattern, with a steep decline in depression risk at lower CALLY values and a plateau effect beyond a threshold of approximately 17.75. Furthermore, subgroup analyses revealed that this protective effect was significantly more pronounced in participants younger than 65 years. These findings suggest that the CALLY index, an easily obtainable composite biomarker reflecting systemic inflammation, nutritional status, and immune function, may serve as a clinically valuable tool for depression risk stratification in the COPD population.

The CALLY Index is an innovative metric that comprehensively assesses multiple dimensions including nutritional status, immune function, and inflammation levels (31), with prognostic value documented across diverse conditions including malignancies (31), rheumatoid arthritis (14, 32), and cardiovascular diseases (13). Our findings are broadly consistent with the limited but growing evidence linking the CALLY index to mental health outcomes. A recent cross-sectional study using NHANES data among 3,517 adults with type 2 diabetes mellitus reported that individuals in the highest CALLY quartile had a 42% lower prevalence of depression compared with those in the lowest quartile (OR 0.58, 95% CI 0.38–0.89), and no significant non-linearity was observed (16). Similarly, a prospective study of 179 patients with acute ischemic stroke demonstrated that a lower CALLY index independently predicted post-stroke depression at six months, with an optimal cutoff of 1.09. Our study extends these observations to the COPD population for the first time. Prior investigations of depression in COPD have predominantly focused on individual inflammatory markers rather than composite immune nutritional indices. Strollo et al. found that IL-6, but not CRP or TNF-α, was independently associated with depressive symptoms across two large COPD cohorts (SCCOR and COPD Gene) (33), while Al-shair et al. reported that TNF-α, rather than CRP or IL-6, correlated with depression in moderate COPD (34), and Lu et al. (35) showed that both IL-6 and CRP were positively associated with depressive symptoms and partially mediated the link between pulmonary function and depression. These inconsistent results for individual biomarkers highlight a potential advantage of the CALLY index, which integrates inflammation (CRP), nutrition (albumin), and immunity (lymphocyte) into a single composite measure, thereby more comprehensively capturing the multisystem dysregulation—encompassing systemic inflammation, muscle wasting, and immune senescence—that characterizes COPD and its comorbid depression.

Several distinctive findings of this study warrant detailed consideration. The non-linear dose-response relationship, characterized by a threshold effect at CALLY ≈ 17.75, carries important clinical implications. Below this threshold, each unit increase in CALLY conferred a significant 5% reduction in depression risk, whereas above the threshold the association flattened and became non-significant. This plateau effect suggests that the biological systems linking CALLY to depression risk may become saturated once adequate inflammatory suppression, nutritional repletion, and immune competence are achieved. Clinically, this implies that the greatest mental health benefits may be realized by improving CALLY components in patients with markedly elevated inflammation and poor nutritional status, rather than pursuing extreme biomarker optimization. Importantly, the threshold value identified in this study is a data-driven statistical estimate specific to our cohort and analytical framework. It should not be interpreted as a definitive clinical cut-off for risk stratification. External validation in independent cohorts with diverse demographic and clinical characteristics is necessary before any clinical application of this threshold can be recommended.

The nominally significant age-related heterogeneity observed in subgroup analyses further refines the clinical applicability of these findings. The nominally significant interaction for age (*P* = 0.017), which did not survive Bonferroni correction, with a protective effect in participants < 65 years (HR = 0.91) but not in those ≥65 years (HR = 0.99), suggests that CALLY-based risk stratification may be most informative in middle-aged COPD patients. In younger patients, depression may be more strongly driven by modifiable biological processes such as systemic inflammation and nutritional status, whereas in older adults, depression etiology may be increasingly dominated by neurodegenerative changes, polypharmacy, social isolation, and competing multi-morbidity burdens that are less captured by the CALLY index. Additionally, the wider confidence intervals in the older subgroup, reflecting smaller sample size and fewer events, may have limited statistical power. However, given that this age interaction did not survive correction for multiple comparisons, it should be interpreted as hypothesis-generating rather than confirmatory. These findings suggest that age-specific intervention strategies may be warranted, with CALLY-guided prevention efforts prioritized for younger COPD populations.

Several interconnected biological pathways may explain the observed inverse association between CALLY and depression risk in COPD. Elevated CRP activates the kynurenine pathway via indoleamine 2,3-dioxygenase, diverting tryptophan from serotonin synthesis toward neurotoxic metabolites (36, 37), while pro-inflammatory cytokines impair hippocampal neurogenesis and dysregulate the hypothalamic-pituitary-adrenal axis (38). Reduced albumin limits plasma tryptophan transport to the brain (39) and lower lymphocyte counts reflect altered T-cell homeostasis linked to mood dysregulation (40). In COPD, chronic airway inflammation provides a sustained pro-inflammatory milieu that simultaneously affects all three CALLY components (41). And CALLY may therefore function as a composite readout of this interconnected immune-nutritional dysregulation. However, these mechanistic interpretations remain speculative in the absence of mediation data, and future studies with longitudinal biomarker tracking and formal mediation analysis are needed to confirm these pathways.

The principal strengths of this study include its large-scale prospective design, lengthy follow-up, and comprehensive adjustment for confounders. Multiple sensitivity analyses confirmed result robustness against reverse causation and missing data bias. Competing risk of death, and co-existing respiratory conditions. Furthermore, the UK Biobank's linkage to national hospital and mortality records enabled systematic and standardized ascertainment of depression outcomes. However, several limitations should be acknowledged. First, residual confounding is likely. Despite extensive covariate adjustment, unmeasured factors such as unmeasured COPD severity indicators (e.g., GOLD stage, exacerbation history, mMRC score), diet quality, healthcare access, and health literacy may still bias the observed association. Second, COPD was defined using both spirometry and self-reported diagnosis, which introduces phenotypic heterogeneity and potential misclassification; the absence of systematic post-bronchodilator data and GOLD staging further limits severity characterization. Third, depression ascertainment via ICD-10 codes likely underestimates true incidence, as milder or primary care–managed cases may lack formal diagnostic coding. Fourth, the analytic sample represents a selected minority of the original cohort, and exclusion due to missing spirometry or covariates may be non-random. Additionally, UK Biobank participants are healthier and more socioeconomically advantaged than the general UK population, which may affect generalizability. Fifth, CALLY was derived from single baseline measurements, so intra-individual variation in its components over time remains unaccounted for. Finally, the ethnic homogeneity of the sample (91.3% White) limits inference for non-White populations.

## Conclusion

This prospective cohort study demonstrated that a higher CALLY index was independently associated with a lower risk of incident depression. The association exhibited a significant non-linear pattern. A nominally stronger inverse association was observed in participants younger than 65 years, although this age interaction did not survive correction for multiple comparisons. These findings indicate that the CALLY index may serve as a readily available and clinically interpretable tool for identifying COPD patients at elevated risk of depression, and that integrated management targeting inflammation, nutrition, and immune status could be a promising strategy for depression prevention in this vulnerable population. Further research is warranted to elucidate the mechanistic pathways underlying this association and to evaluate whether interventions that improve CALLY components can reduce depression incidence in COPD patients.

## Data Availability

The data analyzed in this study is subject to the following licenses/restrictions: The UK Biobank data are available to bona fide researchers upon formal application through the UK Biobank Access Management System (https://www.ukbiobank.ac.uk/). Access is granted under strict data-sharing agreements and ethical approvals to protect participant confidentiality and privacy. Individual-level data cannot be made publicly available or deposited in unrestricted repositories due to the informed consent provisions and governance framework governing the UK Biobank resource. This research was conducted under UK Biobank application number 1263482. Researchers who meet the UK Biobank criteria for access can apply for the same dataset; analytical code is available from the corresponding author upon reasonable request. Requests to access these datasets should be directed to Yushan Shi, shiyushan6521@163.com.
