# Correction: Association between the C-reactive protein-albumin-lymphocyte index and incident depression in patients with chronic obstructive pulmonary disease: a prospective cohort study from the UK biobank

**DOI:** 10.3389/fmed.2026.2010397

**Published:** 2026-10-23

**Authors:** Frontiers Production Office

**Affiliations:** Frontiers Media SA, Lausanne, Switzerland

**Keywords:** chronic obstructive pulmonary disease, c-reactive protein–albumin–lymphocyte index, depression, non-linear association, prospective cohort study, threshold effect, UK biobank

The figure captions were in the wrong order in the PDF and online version of this paper. The legends for Figure 3 and Figure 4 were reversed. The order has now been corrected.

The original version of this article has been updated.

