# Supplementary material for: Association between the C-reactive protein-albumin-lymphocyte index and incident depression in patients with chronic obstructive pulmonary disease: a prospective cohort study from the UK biobank
Source: Front Med (Lausanne). 2026 Aug 28;13:1921399. doi: 10.3389/fmed.2026.1921399 (PMC13587836; doi:10.3389/fmed.2026.1921399)
Supplement: Supplementary file 1 [file Data_Sheet_1.PDF]

CALLY Index and Depression Risk in COPD: A Prospective Cohort Study

STUDY POPULATION & DESIGN

UK Biobank

COPD Participants

N = 40,200

- Prospective cohort design
- Median follow-up: 13.1 years
- Incident depression (ICD-10 codes)
- 4 sequential covariate models

Depression Events
2,059 (5.1%)

Sensitivity analyses
Competing risk · MI · RCS · Subgroup · E-value

Cox Regression

CALLY INDEX (EXPOSURE)

Composite Immunonutritional Index

$$CALLY = \frac{\text{Albumin (g/L)} \times \text{Lymphocyte } (10^9/\text{L})}{\text{CRP (mg/L)}}$$

Three Component Dimensions

- **Albumin** → Nutritional status
- **Lymphocyte** → Immune function
- **CRP (inverse)** → Inflammation

Higher CALLY = Better immunonutritional status
(lower inflammation, preserved nutrition/immunity)

↓ Depression Risk

KEY FINDINGS

Per 1-unit increase in ln(CALLY)

Fully adjusted (Model 4)
HR 0.93 (0.89–0.98)

Highest vs Lowest Quartile

HR 0.82
(0.72–0.95)

Trend across quartiles: **P = 0.001**

Age Interaction (P = 0.017)

< 65 years: HR 0.91 (0.87–0.96)
≥ 65 years: HR 0.99 (0.91–1.06)

Dose-response (RCS): **P-nonlinear = 0.022**
Breakpoint CALLY ≈ 1 7 . 7 5
Above breakpoint: HR 0.95 (0.90-0.99)

CONCLUSION

Higher CALLY index is independently associated with lower risk of incident depression
among adults with COPD in the UK Biobank, particularly in those younger than 65 years.

CALLY may serve as a clinically accessible immunonutritional marker for depression risk stratification in COPD.
