# Supplementary material for: Association between the C-reactive protein-albumin-lymphocyte index and incident depression in patients with chronic obstructive pulmonary disease: a prospective cohort study from the UK biobank
Source: Front Med (Lausanne). 2026 Aug 28;13:1921399. doi: 10.3389/fmed.2026.1921399 (PMC13587836; doi:10.3389/fmed.2026.1921399)
Supplement: Supplementary file 2 [file Supplementary_file_1.docx]

**Association Between the C-Reactive Protein-Albumin-Lymphocyte Index and Incident Depression in Patients with Chronic Obstructive Pulmonary Disease: A Prospective Cohort Study from the UK Biobank**

Yushan Shi^1,2^, Xianhai Chen^3*^ , Yan Wang^3*^

^1^First Clinical Medical College, Shandong University of Traditional Chinese Medicine, Jinan, Shandong, China

^2^Department of Laboratory Medicine, Affiliated Hospital of Shandong University of Traditional Chinese Medicine, Jinan, Shandong, China

^3^Department of Respiratory and Critical Care Medicine, Affiliated Hospital of Shandong University of Traditional Chinese Medicine, Jinan, Shandong, China

^*^Corresponding author

Xianhai Chen

Email: [chenxianhai18@163.com](mailto:chenxianhai18@163.com)

Department of Respiratory and Critical Care Medicine, Affiliated Hospital of Shandong University of Traditional Chinese Medicine, Jinan, Shandong, China

Yan Wang

Email: yan02356@163.com

Department of Respiratory and Critical Care Medicine, Affiliated Hospital of Shandong University of Traditional Chinese Medicine, Jinan, Shandong, China

**Table S1. Association between CALLY and incident depression after excluding participants with less than 2 years of follow-up (n=39,732)**

| **Variable** | **Model 1** | |  | **Model 2** | |  | **Model3** | |  | **Model4** | |  | **Model5** | |
| --- | --- | --- | --- | --- | --- | --- | --- | --- | --- | --- | --- | --- | --- | --- |
|  | **HR (95%CI)** | ***p*** |  | **HR (95%CI)** | ***p*** |  | **HR (95%CI)** | ***p*** |  | **HR (95%CI)** | ***p*** |  | **HR (95%CI)** | ***p*** |
| ln-CALLY | 0.84 (0.8~0.87) | <0.001 |  | 0.88 (0.84~0.92) | <0.001 |  | 0.93 (0.89~0.98) | 0.003 |  | 0.94 (0.89~0.98) | 0.004 |  | 0.94 (0.9~0.98) | 0.005 |
| CALLY quartiles |  |  |  |  |  |  |  |  |  |  |  |  |  |  |
| Q1 (<2.91) | 1(Ref) |  |  | 1(Ref) |  |  | 1(Ref) |  |  | 1(Ref) |  |  | 1(Ref) |  |
| Q2 (2.91-6.11) | 0.84 (0.75~0.95) | 0.004 |  | 0.9 (0.8~1.02) | 0.09 |  | 0.96 (0.85~1.09) | 0.559 |  | 0.98 (0.86~1.11) | 0.709 |  | 0.98 (0.87~1.11) | 0.787 |
| Q3 (6.11-12.52) | 0.66 (0.58~0.74) | <0.001 |  | 0.74 (0.65~0.84) | <0.001 |  | 0.82 (0.72~0.94) | 0.004 |  | 0.84 (0.73~0.96) | 0.009 |  | 0.84 (0.73~0.96) | 0.01 |
| Q4 (≥12.52） | 0.59 (0.52~0.67) | <0.001 |  | 0.69 (0.61~0.79) | <0.001 |  | 0.83 (0.72~0.96) | 0.011 |  | 0.84 (0.73~0.97) | 0.017 |  | 0.84 (0.73~0.97) | 0.017 |
| *P* for trend |  | <0.001 |  |  | <0.001 |  |  | 0.002 |  |  | 0.003 |  |  | 0.003 |

**Note:** Model 1: unadjusted.

Model 2: adjusted for age, sex, ethnic background, education level, household income, TDI, and assessment.

Model 3: additionally adjusted for BMI, smoking status, alcohol drinker status, summed MET, Sleep duration, SBP, FVC, and FEV₁.

Model 4: additionally adjusted for WBC, platelet, hemoglobin, glucose, ALT, creatinine, HbA1c, HDL-C, triglycerides, and Urea.

Model 5: additionally adjusted for history of asthma, diabetes, hypertension.

**Abbreviation:** COPD, chronic obstructive pulmonary disease; CALLY, C-reactive protein-albumin-lymphocyte index; TDI, Townsend deprivation index; BMI, body mass index; MET, metabolic equivalent of task; SBP, Systolic blood pressure; FVC, forced vital capacity; FEV1, forced expiratory volume in 1 second; WBC, White blood cell; CRP, C-reactive protein; ALT, Alanine aminotransferase; HbA1c, glycated hemoglobin; HDL, high-density lipoprotein.

**Table S2. Association between CALLY and incident depression after multiple imputation for missing covariates (n=60599)**

| **Variable** | **Model 1** | |  | **Model 2** | |  | **Model3** | |  | **Model4** | |  | **Model5** | |
| --- | --- | --- | --- | --- | --- | --- | --- | --- | --- | --- | --- | --- | --- | --- |
|  | **HR (95%CI)** | ***p*** |  | **HR (95%CI)** | ***p*** |  | **HR (95%CI)** | ***p*** |  | **HR (95%CI)** | ***p*** |  | **HR (95%CI)** | ***p*** |
| ln-CALLY | 0.82 (0.79~0.84) | <0.001 |  | 0.86 (0.84~0.89) | <0.001 |  | 0.92 (0.88~0.96) | <0.001 |  | 0.93 (0.9~0.98) | 0.002 |  | 0.94 (0.9~0.98) | 0.003 |
| CALLY quartiles |  |  |  |  |  |  |  |  |  |  |  |  |  |  |
| Q1 (<2.91) | 1(Ref) |  |  | 1(Ref) |  |  | 1(Ref) |  |  | 1(Ref) |  |  | 1(Ref) |  |
| Q2 (2.91-6.11) | 0.79 (0.72~0.86) | <0.001 |  | 0.84 (0.77~0.92) | <0.001 |  | 0.92 (0.82~1.04) | 0.184 |  | 0.97 (0.86~1.09) | 0.572 |  | 0.97 (0.86~1.1) | 0.671 |
| Q3 (6.11-12.52) | 0.64 (0.58~0.7) | <0.001 |  | 0.72 (0.66~0.79) | <0.001 |  | 0.81 (0.71~0.91) | 0.001 |  | 0.83 (0.73~0.94) | 0.004 |  | 0.83 (0.73~0.95) | 0.006 |
| Q4 (≥12.52） | 0.54 (0.49~0.59) | <0.001 |  | 0.64 (0.58~0.71) | <0.001 |  | 0.77 (0.67~0.88) | <0.001 |  | 0.82 (0.71~0.94) | 0.004 |  | 0.82 (0.71~0.94) | 0.005 |
| *P* for trend |  | <0.001 |  |  | <0.001 |  |  | <0.001 |  |  | 0.001 |  |  | 0.001 |

**Note:** Model 1: unadjusted.

Model 2: adjusted for age, sex, ethnic background, education level, household income, TDI, and assessment.

Model 3: additionally adjusted for BMI, smoking status, alcohol drinker status, summed MET, Sleep duration, SBP, FVC, and FEV₁.

Model 4: additionally adjusted for WBC, platelet, hemoglobin, glucose, ALT, creatinine, HbA1c, HDL-C, triglycerides, and Urea.

Model 5: additionally adjusted for history of asthma, diabetes, hypertension.

**Abbreviation:** COPD, chronic obstructive pulmonary disease; CALLY, C-reactive protein-albumin-lymphocyte index; TDI, Townsend deprivation index; BMI, body mass index; MET, metabolic equivalent of task; SBP, Systolic blood pressure; FVC, forced vital capacity; FEV1, forced expiratory volume in 1 second; WBC, White blood cell; CRP, C-reactive protein; ALT, Alanine aminotransferase; HbA1c, glycated hemoglobin; HDL, high-density lipoprotein.

**Table S3. Fine-Gray competing risk model results**

| **Variable** | **Model 1** | |  | **Model 2** | |
| --- | --- | --- | --- | --- | --- |
|  | **SHR (95%CI)** | ***p*** |  | **SHR (95%CI)** | ***p*** |
| ln-CALLY | 0.86 (0.83~0.89) | <0.001 |  | 0.96 (0.92~0.99) | 0.035 |
| CALLY quartiles |  |  |  |  |  |
| Q1 (<2.91) | 1(Ref) |  |  | 1(Ref) |  |
| Q2 (2.91-6.10) | 0.88 (0.78~0.99) | 0.027 |  | 1.02 (0.9~1.15) | 0.744 |
| Q3 (6.10-12.52) | 0.69 (0.6~0.78) | <0.001 |  | 0.87 (0.76~0.99) | 0.044 |
| Q4 (≥12.52） | 0.63 (0.56~0.72) | <0.001 |  | 0.87 (0.75~0.99) | 0.046 |
| *P* for trend |  | <0.001 |  |  | 0.011 |

**Note:** Model 1: unadjusted.

Model 2: adjusted for age, sex, ethnic background, education level, household income, TDI, assessment, BMI, smoking status, alcohol drinker status, summed MET, Sleep duration, SBP, FVC, FEV₁, WBC, platelet, hemoglobin, glucose, ALT, creatinine, HbA1c, HDL-C, triglycerides, Urea, history of asthma, diabetes, hypertension.

**Abbreviation:** COPD, chronic obstructive pulmonary disease; CALLY, C-reactive protein-albumin-lymphocyte index; TDI, Townsend deprivation index; BMI, body mass index; MET, metabolic equivalent of task; SBP, Systolic blood pressure; FVC, forced vital capacity; FEV1, forced expiratory volume in 1 second; WBC, White blood cell; CRP, C-reactive protein; ALT, Alanine aminotransferase; HbA1c, glycated hemoglobin; HDL, high-density lipoprotein.

**Table S4. Association between CALLY and incident depression after excluding concomitant respiratory diseases (n = 30,526)**

| **Variable** | **Model 1** | |  | **Model 2** | |  | **Model3** | |  | **Model4** | |  | **Model5** | |
| --- | --- | --- | --- | --- | --- | --- | --- | --- | --- | --- | --- | --- | --- | --- |
|  | **HR (95%CI)** | ***p*** |  | **HR (95%CI)** | ***p*** |  | **HR (95%CI)** | ***p*** |  | **HR (95%CI)** | ***p*** |  | **HR (95%CI)** | ***p*** |
| ln-CALLY | 0.85 (0.81~0.89) | <0.001 |  | 0.9 (0.86~0.94) | <0.001 |  | 0.94 (0.89~0.99) | 0.015 |  | 0.94 (0.89~0.99) | 0.022 |  | 0.94 (0.89~0.99) | 0.015 |
| CALLY quartiles |  |  |  |  |  |  |  |  |  |  |  |  |  |  |
| Q1 (<3.04) | 1(Ref) |  |  | 1(Ref) |  |  | 1(Ref) |  |  | 1(Ref) |  |  | 1(Ref) |  |
| Q2 (3.04-6.36) | 0.84 (0.73~0.96) | 0.011 |  | 0.89 (0.78~1.02) | 0.088 |  | 0.93 (0.81~1.07) | 0.301 |  | 0.94 (0.82~1.08) | 0.407 |  | 0.94 (0.82~1.08) | 0.394 |
| Q3 (6.36-12.99) | 0.65 (0.57~0.75) | <0.001 |  | 0.73 (0.63~0.84) | <0.001 |  | 0.79 (0.68~0.92) | 0.002 |  | 0.81 (0.69~0.94) | 0.006 |  | 0.80 (0.69~0.94) | 0.005 |
| Q4 (≥12.99） | 0.62 (0.53~0.71) | <0.001 |  | 0.72 (0.62~0.84) | <0.001 |  | 0.81 (0.69~0.95) | 0.01 |  | 0.82 (0.7~0.97) | 0.017 |  | 0.81 (0.69~0.95) | 0.01 |
| *P* for trend |  | <0.001 |  |  | <0.001 |  |  | 0.002 |  |  | 0.004 |  |  | 0.002 |

**Note:** Model 1: unadjusted.

Model 2: adjusted for age, sex, ethnic background, education level, household income, TDI, and assessment.

Model 3: additionally adjusted for BMI, smoking status, alcohol drinker status, summed MET, Sleep duration, SBP, FVC, and FEV₁.

Model 4: additionally adjusted for WBC, platelet, hemoglobin, glucose, ALT, creatinine, HbA1c, HDL-C, triglycerides, and Urea.

Model 5: additionally adjusted for history of diabetes, hypertension.

Quartile cutoffs were calculated within this subpopulation (n = 30,526) and therefore differ slightly from those in the primary analysis.

**Abbreviation:** COPD, chronic obstructive pulmonary disease; CALLY, C-reactive protein-albumin-lymphocyte index; TDI, Townsend deprivation index; BMI, body mass index; MET, metabolic equivalent of task; SBP, Systolic blood pressure; FVC, forced vital capacity; FEV1, forced expiratory volume in 1 second; WBC, White blood cell; CRP, C-reactive protein; ALT, Alanine aminotransferase; HbA1c, glycated hemoglobin; HDL, high-density lipoprotein.

**Table S5.Net reclassification improvement and integrated discrimination improvement for CALLY versus CRP alone in predicting incident depression**

| **Index** | **Estimate(95% CI)** | ***p*** |
| --- | --- | --- |
| NRI(Continuous) | 0.0844(0.042-0.127) | <0.001 |
| IDI | 0.0001(0–0.0002) | 0.077 |

**Note:** NRI, net reclassification improvement; IDI, integrated discrimination improvement; CALLY, C-reactive protein–albumin–lymphocyte index; CRP, C-reactive protein.

**Figure Legends**

**Figure S1.** Receiver operating characteristic (ROC) curves comparing the predictive performance of the C-reactive protein–albumin–lymphocyte (CALLY) index, C-reactive protein (CRP), albumin, and lymphocyte count for incident depression in patients with chronic obstructive pulmonary disease.

AUC, area under the curve; CI, confidence interval.
